# N-acetyl cysteine as an additive to bone cement against pathogens involved in periprosthetic joint infections

**DOI:** 10.3389/fbioe.2025.1595821

**Published:** 2025-08-28

**Authors:** Maja Charlotte Bohn, Hilke Oltmanns, Heidi Harting, Jessica Meißner

**Affiliations:** Department of Pharmacology, Toxicology and Pharmacy, University of Veterinary Medicine Hannover, Hanover, Germany

**Keywords:** N-acetyl cysteine, bone cement, periprosthetic joint infections, antibacterial, cytocompatibility

## Abstract

Periprosthetic joint infections (PJIs) and septic loosening of implants are common complications following surgical replacement of destructive joints in both human and veterinary medicine. Increasing occurrence of multi-resistant bacteria and failure to manage periprosthetic joint infections make it necessary to identify new antibacterial substances for the treatment and prevention of these infections. N-acetyl cysteine (NAC), a derivative of the amino acid cysteine, has been chosen as a candidate substance due to its shown antibacterial activity. The aim of the study was to evaluate the suitability of NAC for the use together with polymethylmethacrylate bone cement in the context of PJIs. Antibacterial activity of pure NAC and NAC-containing bone cement against clinical isolates of *Staphylococcus (S.) pseudintermedius* was tested by determining minimal inhibitory concentrations, analyzing growth of bacteria on bone cement, and examining the influence on infection of human osteosarcoma (HOS) cells. Cytotoxicity of pure NAC and bone cement with NAC against HOS cells was analyzed with viability and proliferation assays, Live/Dead staining of cells on bone cement, measurement of Interleukin-6 (IL-6) release, and visualizing activation of p38 MAP kinase with Western blotting. NAC inhibited growth of *Staphylococcus pseudintermedius* at 2.5 mg/mL and reduced bacterial growth on bone cement but could not inhibit infection of cells at 1.5 mg/mL. The IC_50_ of pure NAC for viability was 3.6 mg/mL. Bone cement with NAC reduced viability and proliferation at some concentrations but did not provoke IL-6 release. Western blots indicated that p38 could be activated following treatment with NAC. Taken together, antibacterial effectiveness could be shown but cytocompatibility of NAC in bone cement was limited, so that NAC cannot currently be used as a bone cement additive. Further research is necessary to balance antibacterial activity and cytotoxicity.

## 1 Introduction

Endoprosthetic replacement of destructive joints is a common procedure in human and veterinary medicine. In 2022, more than 300,000 human primary knee and hip arthroplasties were undertaken in Germany (Grimberg et al., 2023). In veterinary medicine, joint replacement is commonly established as a treatment of joint disease in small animals (Allen, 2012). A complication frequently occurring after total joint arthroplasty is the loosening of prosthetic joints. Next to aseptic loosening due to implant debris, septic loosening caused by infection at the implant site is a common reason for implant failure and revision surgery. Around 15% of revision cases of human patients in Germany were due to infection (Grimberg et al., 2023). The incidence for post-operational infections in dogs varies from 2.6%–10% (Weese, 2008; Hayes et al., 2013). Periprosthetic joint infections (PJIs) are caused by both virulent and opportunistic pathogens and can manifest directly after implantation or weeks later through different routes of infection (Benito et al., 2019). Virulent pathogens causing PJIs are *Staphylococcus aureus* (Benito et al., 2019; Gatti et al., 2022), *Enterococcus* (Renz et al., 2019; Thompson et al., 2019), *Streptococcus* and Gram-negative *Bacillus* species (Bemer et al., 2014). Coagulase-negative *Staphylococcus* species frequently serve as opportunistic pathogens (Benito et al., 2019; Gatti et al., 2022). In small animals, more than 50% of PJIs are caused by S*. aureus* and *Staphylococcus pseudintermedius* (Hayes et al., 2013). *S. pseudintermedius* is a coagulase-positive opportunistic pathogen that colonizes mainly cats and dogs and is responsible for skin infections in these species (Devriese et al., 2005; Perreten et al., 2010). Infection with *S. pseudintermedius* can also occur in humans (Van Hoovels et al., 2006). To prevent the occurrence of PJIs, systemic administration of antibiotics at the time of surgery and fastening of the implant with antibiotic-loaded bone cement is a widespread procedure (Bratzler et al., 2013). Treatment of PJIs includes local antibiotic treatment, debridement of the infection site and replacement of the failed implant. However, the increasing occurrence of antibiotic resistance represents a global threat for human and veterinary medicine in general and challenges the management of PJIs. Resistances are acquired and spread in places with high consumption of antibiotics, e.g., in hospitals, in agriculture and the environment (Almagor et al., 2018; Skandalis et al., 2021). Bacterial mechanisms for defense relevant for PJIs include biofilm formation and quicker adaptation to antibiotic treatment due to increased mutation rate under stress (Swings et al., 2017; Hamad et al., 2022). These problems underline the need for novel treatments in the management of PJIs. Recently proposed alternatives to antibiotic treatment are the use of novel antibiotics or the repurposing of established drugs, antibody therapy and biofilm-disrupting agents (Hamad et al., 2022).

NAC, a derivative of the amino acid cysteine, is commonly used as a mucolytic agent and as an antidote in paracetamol overdose (Plumb, 2018). Its antibacterial activity has been the focus of research in recent years. NAC has been shown to inhibit growth of planktonic bacteria and the formation of biofilms. Furthermore, it can disrupt biofilms alone and in combination with antibiotics, likely due to its acidity and interference with bacterial exopolysaccharide formation (Quah et al., 2012; Manoharan et al., 2020; Chlumsky et al., 2021; Valzano et al., 2022). The thiol group of the molecule can disrupt disulphide bonds of bacterial enzymes, interfering with their function for metabolism and growth (Quah et al., 2012). Antioxidant properties of NAC further contribute to its anti-infective effect (Yamada et al., 2011). NAC can function as a scavenger of ROS extracellularly with its thiol group and as an intracellular supply of glutathione, a powerful cellular antioxidant molecule (Raftos et al., 2007; Yamada et al., 2011). Through the reduction of oxidative stress, NAC can enhance cellular regeneration in the bone (Yamada et al., 2019). An initiatory *in vitro* study has shown that NAC mixed into bone cement exhibits inhibitory effects against planktonic *S. aureus* and *Escherichia coli* and against biofilms (Sukhonthamarn et al., 2021). Although these studies do not suggest that the growth-inhibitory and antibiofilm activity of NAC is species-specific, there is evidence that it could have a beneficial effect on the gut microbiome in diseased patients. In piglets infected with Porcine Epidemic Diarrhea Virus, NAC counteracted severe dysbiosis in the gut microbiome (Wu et al., 2021) and it alleviated gut dysbiosis in mice with metabolic syndrome (Zheng et al., 2019). These effects are mainly based on the antioxidant properties and reduction of pathogenic invasion (Zheng et al., 2019; Wu et al., 2021). Next to its beneficial effects, it is possible that bacteria could develop resistance against NAC. Bacteria isolated from a water environment have been shown to decompose NAC with a variety of enzymes (Kregiel et al., 2019). NAC could also enhance resistance of *Edwardsiella tarda* to different antibiotics by providing intracellular glutathione, enhancing antibiotic efflux, and promoting protein aggregation (Guo et al., 2024).

Bone cement made of polymethylmethacrylate (PMMA) is widely used in orthopedics. The main function of bone cement is to fasten joint implants, but it is also used to restore fractures and to stabilize vertebrae in the treatment of atlantoaxial instability (Hamajima et al., 2020; Tabanez et al., 2021). PMMA bone cements are established as local carriers of antibiotics in endoprosthetics and as beads on a string that can be implanted into the site of infection (Hayes et al., 2013). Incorporated substances are released from pores in the surface layer of PMMA bone cement, with a first “burst” phase with release of high concentrations and following low and stable release (Sun et al., 2021). The most commonly used antibiotics in bone cement are aminoglycosides (e.g., gentamicin) and aminopeptides (Cyphert et al., 2018). An advantage of such antibiotic-laden bone cements (ALBCs) is that high local concentrations of antibiotics can be achieved (Tootsi et al., 2022). However, the use of ALBC has been discussed critically as evidence for its reducing effect on infection rate is limited (Hoskins et al., 2020; Sebastian et al., 2020).

With these challenges in the management of periprosthetic joint infections, it is necessary to identify new antibacterial substances for the use with bone cement. New bacterial resistances need to be avoided to not further limit treatment options. The aim of this study is to give an evaluation of the suitability of NAC together with bone cement as a prevention and treatment option in human and veterinary medicine.

## 2 Materials and methods

### 2.1 Cell line and culture media

The human osteosarcoma cell line HOS (CRL-1543), obtained from the American Type Culture Collection (ATCC, Manassas, USA), was used for the determination of cytotoxicity, bacterial adhesion and internalization. Cells were subcultured twice a week and kept in Eagle’s Minimum Essential Medium with Earle’s balanced salts (EMEM; Carl Roth, Karlsruhe, Germany) with 10% fetal bovine serum (FBS; Bio&Sell, Feucht, Germany), 1% l-glutamine (GIBCO, Paisley, UK), 1% non-essential amino acids (Carl Roth, Karlsruhe, Germany) and 1% penicillin/streptomycin (GIBCO, Grand Island, USA) in an incubator at 37 °C and 5% CO_2_.

### 2.2 Bacterial strains

For the microbiology experiments, two multiresistant isolates (RSP1 and RSP2) and one sensible (SP1) isolate of *S. pseudintermedius* were used. Isolates were taken from dogs in the Clinic for Small Animals and identified and assessed for resistances in the Institute for Microbiology, both University of Veterinary Medicine Hannover, Germany. Bacterial stocks were kept in cryomedium with 80% glycerin at −80 °C and were subcultured on Columbia sheep blood agar (Oxoid Deutschland, Wesel, Germany) at 37 °C overnight prior to the experiments. During experiments, bacteria were cultured in Mueller Hinton bouillon (Sifin Diagnostics, Berlin, Germany) at 37 °C.

### 2.3 Bone cement

PALACOS^®^ LV bone cement (Heraeus Medical, Wehrheim, Germany) was mixed under sterile conditions, and the mixture was filled into silicone molds to produce round platelets with a diameter of 1 cm. NAC (Sigma-Aldrich Chemie, Steinheim, Germany) was previously added to the powder component of the bone cement. Three groups of bone cement platelets were prepared: platelets without any additive (control), one group with 50 mg NAC per platelet, and one group with 100 mg NAC per platelet.

### 2.4 Determination of antibacterial activity

Minimal inhibitory concentrations (MICs) of NAC for the three clinical isolates of *S. pseudintermedius* were determined with broth microdilutions. NAC was dissolved in Mueller Hinton Bouillon and diluted to concentrations of 0.5 mg/mL to 5 mg/mL. A bacterial suspension with a turbidity of 0.5 McFarland was prepared in 0.9% NaCl solution (B. Braun, Melsungen, Germany). NAC solution and bacterial suspension were added to a U-bottom 96-well microtiter plate along with a negative control and a growth control. Inhibition of bacterial growth was evaluated through optical screening for bacterial pellets after 18 h of incubation at 37 °C according to CLSI standard (CLSI, 2012). The influence of NAC on bacterial growth on bone cement was tested by seeding 5*10^6^ colony forming units per mL (cfu/mL) RSP1 onto the surface of NAC-containing bone cement platelets for 24 h and afterwards staining with the LIVE/DEAD™ BacLight™ Bacterial Viability Kit (Thermo Fisher Scientific, Waltham, USA) according to the manufacturer’s protocol. Live and dead bacterial cells were then examined by fluorescence microscopy (BZ-X800 Inverted Fluorescence-Phase Contrast-Microscope, Keyence Corporation, Osaka, Japan) at 470 nm and 545 nm excitation and 525 nm and 605 nm emission, respectively.

### 2.5 Measurement of cell viability and proliferation

Cytotoxic concentrations of NAC were determined measuring the viability and proliferation of HOS cells. For that several concentrations of NAC dissolved in cell culture medium were prepared and cells were treated with the solution for 24 h. Viability was measured with neutral red assays and proliferation was tested in crystal violet assays. Cells were grown to confluency prior to viability testing or were seeded 4 hours prior to proliferation testing. Cytotoxicity of bone cement was tested by measuring cell viability and proliferation after treatment with bone cement supernatants. Bone cement platelets with 50 mg NAC, 100 mg NAC, and without additive (control) were used and each type of platelet was incubated in 1, 3, 10 and 30 mL cell culture medium for 24 h. HOS cells were treated (72 h after seeding for viability testing, 4 hours after seeding for proliferation testing) with the supernatants, 1 μg/mL lipopolysaccharide (LPS) from *E. coli* O55:B5, and 50 μg/mL peptidoglycan (PGN) (as positive controls for Enzyme-linked Immunosorbent Assay (ELISA)) for 24 h, according to the standards for cytotoxicity testing defined in ISO 10993-5; 2009-06 (International Organization for Standardization, 2009). After treatment, the cell supernatants were collected for ELISAs and both cell viability and proliferation were measured. Growth of HOS cells on the surface of bone cement platelets supplemented with NAC was analyzed by staining the cells with the Live/Dead™ Viability/Cytotoxicity Kit (Thermo Fisher Scientific, Waltham, USA) according to the manufacturer’s protocol and visualizing live and dead cells with fluorescence microscopy (BZ-X800 Inverted Fluorescence-Phase contrast-Microscope, Keyence Corporation, Osaka, Japan) at 470 nm and 545 nm excitation and 525 nm and 605 nm emission, respectively. Ten sections of every platelet with a size of 2.2 mm^2^ each were photographed and cell numbers in each photo were counted with the Keyence BZ-X800 Analyzer software (Keyence Corporation, Osaka, Japan). The mean number of living or dead cells in the ten sections of every platelet and the cell number per mm^2^ were then calculated.

### 2.6 Enzyme-linked Immunosorbent Assay (ELISA)

Release of Interleukin-6 (IL-6) in the samples form HOS cells incubated with bone cement supernatants was measured with DuoSet^®^ ELISA kit for human IL-6 (R&D Systems, Minneapolis, USA). The ELISA was performed according to the manufacturer’s instructions. Optical density of samples and standards was measured at 450 and 570 nm with a MRX microplate reader (Dynex Technologies, Chantilly, USA). Cytokine concentration was calculated with a standard curve generated with Microsoft Excel.

### 2.7 Western blotting

HOS cells were treated for 30 min with 3, 4, or 5 mg/mL NAC or bone cement with 50 mg NAC, 100 mg NAC or without additive incubated in 3 mL medium. After the treatment, cells were washed twice with ice-cold phosphate-buffered saline (PBS) and lysed in radioimmunoprecipitation assay (RIPA) buffer containing protease (cOmplete™ ultra mini EDTA free, Roche, Mannheim, Germany) and phosphatase inhibitors (PhosSTOP™, Roche, Mannheim, Germany). Lysates were centrifuged at 15,000 *g* and 4 °C and the protein-containing supernatants were taken and stored at −80 °C until use. Total protein content in all supernatants was determined using Pierce™ BCA Protein Assay Kit (Thermo Scientific, Rockford, USA). Based on the results of this assay, sample aliquots were adjusted to 10 µg protein per 10 µL and mixed with Laemmli sample buffer (with 25% glycerol (Carl Roth, Karlsruhe, Germany), 5% sodium dodecyl sulfate, 5% β-mercaptoethanol (Sigma-Aldrich Chemie, Steinheim, Germany), 150 mM Tris pH 6.8, 0.05% bromophenol blue (Merck KGaA, Darmstadt, Germany). Proteins were separated using 10% Mini-PROTEAN TGX Precast Protein gels (Bio-Rad, Feldkirchen, Germany) and then blotted onto nitrocellulose membranes (Cytiva Amersham™, Little Chalfont, UK). Total protein blotted onto membranes was stained with Ponceau S Red (Carl Roth, Karlsruhe, Germany; 0.01% in 1% acetic acid) and the total protein image was taken (ChemiDoc MP Imaging System, Bio-Rad, Feldkirchen, Germany) before destaining the membranes and blocking in nonfat milk for 2 h at room temperature. Membranes were washed with Tris-buffered saline with 1% Tween-20 (TBS-T) after each incubation step. Primary antibodies used were p38 MAPK Polyclonal Antibody (AHO1202, Thermo Fisher Scientific, Waltham, USA), 1:1000 and phospho-p38 MAPK (Thr180, Tyr182) Polyclonal Antibody (44-684G, Thermo Fisher Scientific, Waltham, USA) 1:1000 in 5% BSA in TBS-T. The secondary antibody used was Goat-anti rabbit IgG (H + L) Secondary Antibody, HRP conjugated (31,466, Thermo Fisher Scientific, Waltham, USA) 1:20,000 in nonfat milk. For the detection of protein bands, membranes were incubated with SuperSignal West Pico PLUS chemiluminescence substrate (Thermo Fisher Scientific, Waltham, USA) and imaged with the ChemiDoc MP Imaging System. Total protein normalization and image analysis was carried out using ImageLab software (Bio-Rad, Feldkirchen, Germany).

### 2.8 Influence of NAC on bacterial adhesion and internalization into HOS cells

HOS cells were seeded into 24-well culture plates at 40,000 cells per well and grown to confluency in antibiotic-free cell culture medium. Suspension of the susceptible isolate SP1 was prepared in 0.9% sterile sodium chloride solution to a density of 0.5 McFarland units and diluted to create a MOI (multiplicity of infection) of 10:1. HOS cells and *S. pseudintermedius* were co-incubated together with 1.5 mg/mL NAC of cell culture medium only for two or 4 hours. After co-incubation, cells were washed with sterile PBS and then incubated with cell culture medium containing 100 μg/mL gentamicin for 2 hours to kill extracellular bacteria or incubated with antibiotic-free cell culture medium to keep adherent bacteria. HOS cells were then scraped off the well bottom, collected, centrifuged, and lysed by osmotic shock. Lysis was aided by pipetting the cell suspension up and down with syringe and needle. Lysates were diluted in PBS, plated onto Columbia agar containing 5% sheep blood (Oxoid Deutschland, Wesel, Germany) and incubated overnight at 37 °C. Determination of colony forming units in the lysates was done by counting colonies after 24 h.

### 2.9 Statistical analysis

Statistical analysis was conducted with GraphPad Prism 9 (GraphPad Software, Boston, USA). Non-parametric data was assessed for statistical significance with Kruskal–Wallis tests with Dunn’s multiple comparison or Friedman test, parametric data was assessed with Student’s t-test. Results with p-values ≤0.05 were considered statistically significant: *p ≤ 0.05, **p ≤ 0.01, ***p ≤ 0.001. Analyzed data include at least six biological replicates (with the only exception of the PGN positive control in the ELISA results).

## 3 Results

### 3.1 Antibacterial activity

Minimal inhibitory concentrations (MICs) of NAC against two multiresistant isolates and one sensible isolate of *S. pseudintermedius* determined in broth microdilutions demonstrated that NAC had a clear inhibitory effect on all three isolates at 2.5 mg/mL.

**FIGURE 1:**
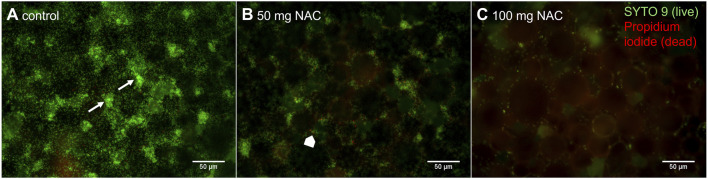
Representative image of growth of *Staphylococcus pseudintermedius* (RSP1) on bone cement. *S. pseudintermedius* were seeded on bone cement platelets and stained with SYTO 9 (green) to visualize live bacteria and propidium iodide (red) to visualize dead bacteria. **(A)**: Bone cement without additive (control), white arrows point to groups of mostly live bacteria growing around bone cement particles. **(B)**: Bone cement with 50 mg NAC, the white block arrow points to dead bacterial cells located in a group of live bacteria. **(C)**: Bone cement with 100 mg NAC. Scale bar: 50 µm.

For incorporation of NAC into bone cement, 50 mg and 100 mg per platelet were chosen to reach antibacterial concentrations. Bacterial attachment and growth (Figure 1) were observed mostly around PMMA polymer particles, as pointed out by the arrows in Figure 1A. On bone cement without additive, intensive green staining indicated that *S. pseudintermedius* was able to attach to the cement and stay viable on its surface (Figure 1A). Adding 50 mg of NAC to the bone cement (Figure 1B) lead to a lower staining intensity compared to cement without additive, which correlates with less attached bacteria on the cement. Furthermore, a higher portion of bacterial cells was dead. Least attachment and growth were observed on bone cement with 100 mg NAC (Figure 1C). Thus, the ability of *S. pseudintermedius* to attach to and grow on bone cement decreases with rising NAC concentrations.

### 3.2 Influence on cell viability, proliferation, and cytokine release

**FIGURE 2:**
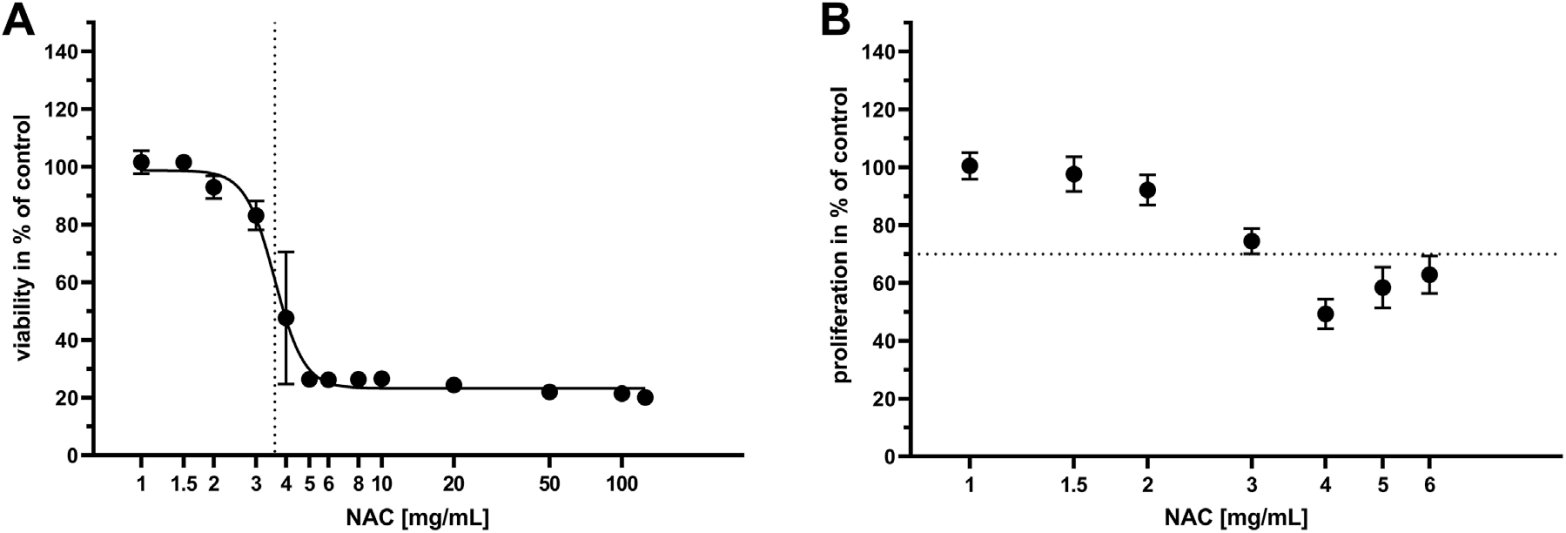
Influence of different NAC concentrations on viability and proliferation of HOS cells. Each value is the mean of at least six independent experiments (SD). Control equals treatment with cell culture medium only. **(A)**: Dose-response graph of 0–125 mg/mL NAC for viability of HOS cells. The x-axis shows NAC concentration, the y-axis shows the cell viability in percent of control. The dotted line indicates the half-maximal inhibitory concentration (IC_50_), which is 3.6 mg/mL NAC. **(B)**: Dose-response graph of 0–6 mg/mL NAC for proliferation of HOS cells. The x-axis shows NAC concentration, the y-axis shows cell proliferation in percent of control. The dotted line represents the 70% limit for cytotoxicity testing according to ISO 10993-5; 2009-06 (International Organization for Standardization, 2009).

Similar to the determination of antibacterial concentrations, concentrations of NAC with a cytotoxic effect on HOS cells were identified. Viability and proliferation of cells after treatment with NAC were assessed and the results are displayed in dose-response curves (Figure 2). Viability of HOS cells (Figure 2A) was higher than 70% of control at 1–3 mg/mL, referring to the 70% viability limit for cytotoxicity testing according to ISO 10993-5; 2009-06 (International Organization for Standardization, 2009). The half-maximal inhibitory concentration (IC_50_) was 3.6 mg/mL. All higher tested concentrations had a cytotoxic effect. Following the results for viability testing, 1–6 mg/mL NAC were chosen for HOS cell proliferation testing. At concentrations higher than 3 mg/mL, proliferation was lower than 70% of control (Figure 2B) so that NAC was considered cytotoxic for HOS cells at these concentrations.

**FIGURE 3:**
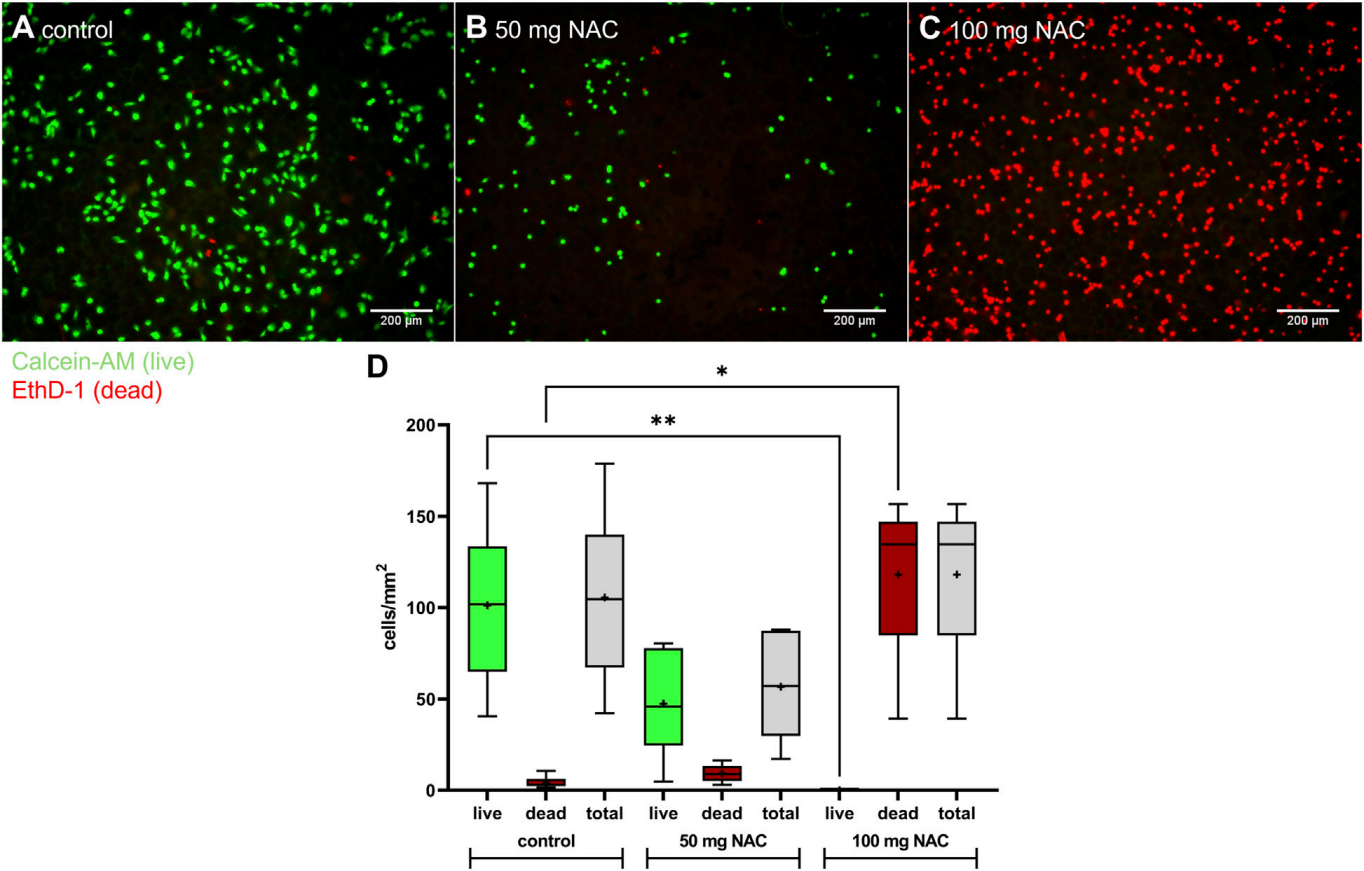
Growth of HOS cells on bone cement platelets containing NAC. **(A**–**C)**: Live/Dead staining of HOS cells seeded on bone cement platelets without additive (A; control), 50 mg NAC **(B)**, 100 mg NAC **(C)**. Live cells are stained with Calcein-AM (green) and dead cells are stained with ethidium homodimer-1 (EthD-1; red). Scale bar: 200 µm. **(D)**: Live and dead HOS cells per mm^2^ on bone cement platelets without additive, 50 mg NAC, and 100 mg NAC (n = 6 for each concentration). Data are shown as boxplots with min/max-whiskers and the mean is depicted as +. Kruskal–Wallis test with Dunn’s multiple comparison test; *p ≤ 0.05, **p ≤ 0.01; asterisks indicate significant differences between numbers of live cells or dead cells.

**FIGURE 4:**
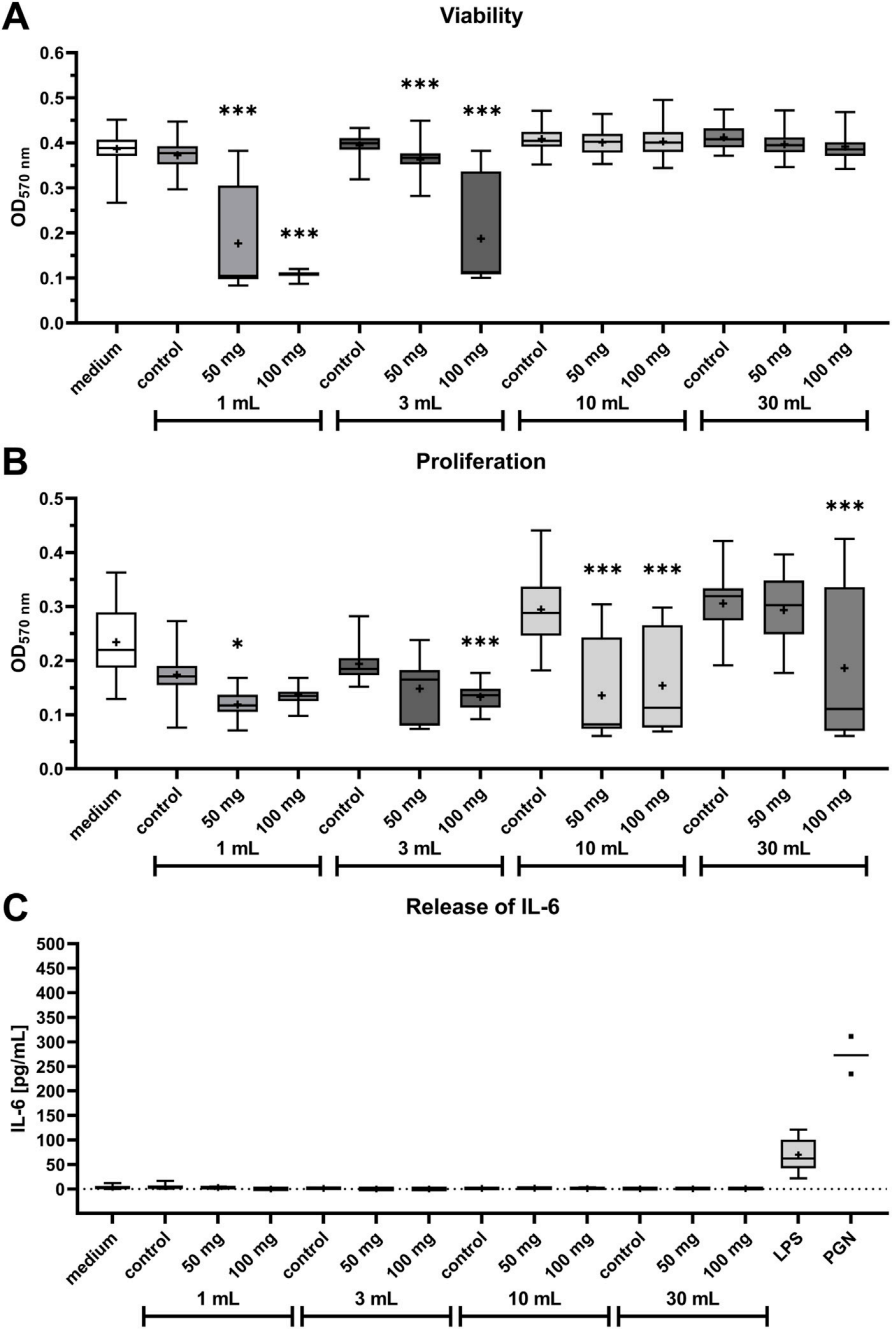
Influence of NAC-containing bone cement incubated in four different volumes of cell culture medium on viability, proliferation, and Interleukin-6 (IL-6) release of HOS cells. **(A,B)**: Viability and proliferation of HOS cells after 24 h of treatment with supernatants of bone cement platelets without additive (control), 50 mg NAC, and 100 mg NAC. The y-axis shows the optical density measured in the viability/proliferation assay; data are shown as boxplots with min/max-whiskers with the mean depicted as + (n ≥ 6 for every treatment). Kruskal–Wallis test with Dunn’s multiple comparison test; *p ≤ 0.05, **p ≤ 0.01, ***p ≤ 0.001; asterisks indicate significant differences in comparison to the sample’s respective control. **(C)**: Release of IL-6 (in pg/mL) from HOS cells after 24 h of treatment with supernatants of bone cement platelets without additive (control), 50 mg NAC, 100 mg NAC, 1 μg/mL lipopolysaccharide (LPS) B5, and 50 μg/mL peptidoglycan (PGN). LPS and PGN treatment served as positive control. Data are shown as boxplots with min/max-whiskers and the mean is depicted as + (n = 2 for PGN; all other treatments n ≥ 6). No significant differences were found between cells treated with NAC-containing supernatants and cells treated with supernatants without additive.

After characterizing the effect of pure NAC on HOS cells, further experiments were conducted with bone cement with 50 mg, 100 mg NAC, and without additive (control). Attachment and growth of HOS cells in direct contact with NAC-containing bone cement (Figure 3) was examined as well as viability, proliferation and release of IL-6 were tested after treatment of HOS with NAC-containing bone cement supernatants (Figure 4).

For direct contact, HOS were seeded onto bone cement platelets. On bone cement without additive (Figure 3A), cells were able to attach to the cement and began to regain their usual stretched morphology. Only a few dead cells (red, stained with ethidium homodimer-1) could be seen between live cells (green, stained with calcein-AM). With 50 mg NAC (Figure 3B), there was a lower number of cells attached to the cement compared to control, while with 100 mg NAC (Figure 3C), all attached cells were dead. Total cell number per mm^2^ (Figure 3D) on platelets with 50 mg NAC was lower compared to control and compared to 100 mg NAC, but not statistically significant. On average, there were 105 cells/mm^2^ on control bone cement without additive, 56 cells/mm^2^ on bone cement with 50 mg NAC, and 118 cells/mm^2^ on bone cement with 100 mg NAC. Since there were no live cells on bone cement platelets with 100 mg NAC, there were significantly less live cells compared to the control (on average 101 live cells/mm^2^). In contrast, the number of dead cells per mm^2^ was significantly higher with 100 mg NAC (118 cells/mm^2^) compared to control (4 cells/mm^2^). At the tested concentrations, NAC hindered HOS cells from growing on the cement and exhibited a cytotoxic effect.

Supernatant of bone cement with 50 mg NAC and 100 mg NAC in 1 mL and 3 mL reduced cell viability significantly compared to bone cement without additive (Figure 4A). Treatment with 10 mL or 30 mL supernatants did not reduce viability. Proliferation of HOS cells was more strongly affected by treatment with bone cement supernatants than viability. As seen in Figure 4B, proliferation was significantly reduced by bone cement with 50 mg NAC in 1 mL and 10 mL and 100 mg NAC in 3 mL, 10 mL, and 30 mL. Samples for ELISA testing of IL-6 release were collected after the 24 h treatment for viability testing. In the samples, no release of IL-6 could be measured except in the positive controls treated with LPS and PGN and there was no difference between the NAC-containing supernatants and controls (Figure 4C).

### 3.3 p38 MAPK activation

**FIGURE 5:**
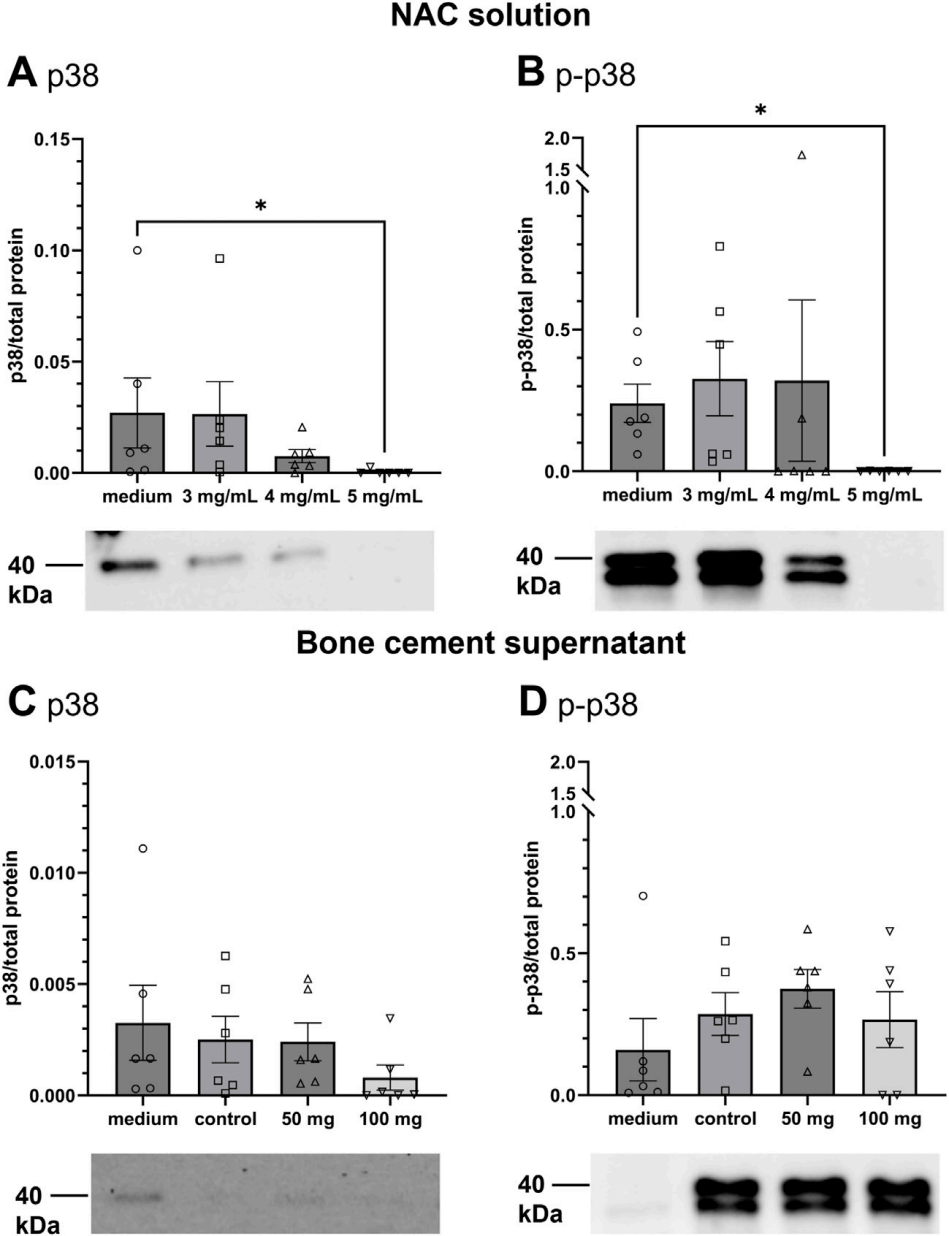
Detection of p38 and p-p38 MAPK in HOS cells after treatment with NAC solution or NAC-containing bone cement supernatants. Graphs show quantification of protein band intensities normalized to total protein; photos depict representative western blots. Protein size is expressed in kDA. **(A,B)**: Levels of p38 **(A)** and p-p38 **(B)** in HOS cells after treatment with 3, 4, and 5 mg/mL NAC in cell culture medium. **(C,D)**: Levels of p38 **(C)** and p-p38 **(D)** after treatment with supernatants of bone cement platelets without additive (control), 50 mg NAC, and 100 mg NAC. All data are shown as mean (n = 6) (standard error of the mean (SEM)), individual data points are shown. Friedman test with Dunn’s post test, *p ≤ 0.05.

We analyzed levels of p38 and its phosphorylated form (p-p38) after contact with NAC and NAC-containing bone cement supernatants. Figure 5 shows the levels of p38 and p-p38 normalized to total lane protein and representative blots. The ratio of p38 to total protein decreased with rising NAC concentrations. It was significantly lower compared to cell culture medium treatment after treatment with 5 mg/mL NAC, with mean ratios of 0.027 and 0.0004, respectively (Figure 5A). Levels of p-p38 (Figure 5B) were slightly raised by treatment with 3 mg/mL and 4 mg/mL NAC. Treatment with 5 mg/mL NAC significantly reduced the p-p38 level. Figure 5C shows levels of p38 after treatment of HOS cells with bone cement supernatants. All bone cement supernatants reduced the level of p38, with 100 mg NAC having the strongest effect, but there was no statistical significance. Levels of p-p38 (Figure 5D) were increased after treatment with bone cement supernatants with the highest increase provoked by 50 mg NAC, but there was also no statistical significance. To conclude this, NAC treatment does seem to influence p38 activation and alter protein levels in HOS cells by increasing p38 activation, but this effect can sometimes be on non-significant levels.

### 3.4 Bacterial adhesion and internalization

**FIGURE 6:**
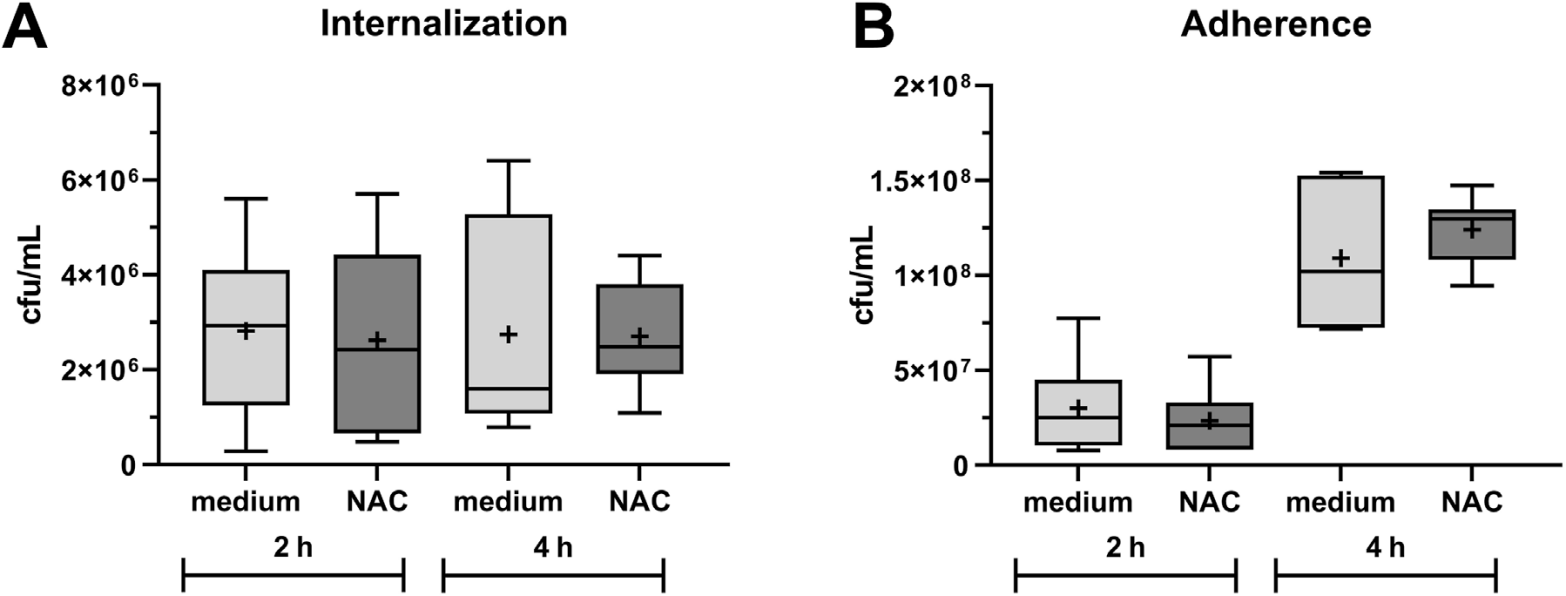
Influence of 1.5 mg/mL NAC on internalization and adherence of *Staphylococcus pseudintermedius* (SP1) into/to HOS cells. Data are shown as boxplots with min/max-whiskers and the mean depicted as + (n = 6 each). **(A)**: Internalized bacteria in colony forming units (cfu) per mL after 2 h and 4 h co-incubation of HOS cells, *Staphylococcus pseudintermedius*, and NAC or cell culture medium. **(B)**: Bacteria adherent to HOS cells in cfu per mL after 2 h and 4 h of co-incubation of HOS, *Staphylococcus pseudintermedius*, and NAC or cell culture medium. Student’s t-tests did not reveal any significant differences between NAC and cell culture medium treatment.

The internalization of *S. pseudintermedius* SP1 into HOS cells and adherence to the cells in the presence of 1.5 mg/mL NAC was analyzed compared to medium without NAC. The cells were infected for 2 h and 4 h. Figure 6 shows the count of internalized and adherent bacteria in colony forming units per mL (cfu/mL). The count of internalized *S. pseudintermedius* (Figure 6A) did not differ between NAC or medium treatment or between 2 h and 4 h of infection, with a mean bacterial count of 3*10^6^ cfu/mL in all samples. More bacteria were adherent to HOS cells after 4 h of infection than after 2 h (Figure 6B), with a mean of 1*10^8^ cfu/mL vs 3*10^7^ cfu/mL, but there was no difference between NAC treatment and medium. At 1.5 mg/mL, NAC could not inhibit infection of HOS cells by *S. pseudintermedius*.

## 4 Discussion

Periprosthetic joint infections are one of the leading causes of implant failure. With antibiotic resistances as a major health threat in general, novel prevention methods and treatments for PJIs are needed. In this *in vitro* study, the suitability of NAC as an additive to bone cement against PJI-related pathogens was investigated. NAC presents activity against planktonic bacteria and the ability to prevent the formation of bacterial biofilms in several species (Olofsson et al., 2003; Yamada et al., 2011; Quah et al., 2012; Costa et al., 2017). It is also a promising candidate for combination treatment with antibiotics against mature biofilms (Manoharan et al., 2020; Valzano et al., 2022). Concerning its cytotoxicity, NAC has been shown to be safe for osteoblasts at concentrations up to 1.15 mg/mL in an *in vitro* context (Tsukimura et al., 2009; Yamada et al., 2019). In this study, we demonstrated that NAC inhibits growth of planktonic *S. pseudintermedius* at 2.5 mg/mL and reduced the ability of *S. pseudintermedius* to attach to and grow on bone cement in a dose-dependent manner, but could not prevent infection of HOS cells at a concentration below the MIC. NAC was not cytotoxic for HOS cells at concentrations up to 3 mg/mL, but 50 mg and 100 mg NAC per bone cement platelet impaired cell growth and viability directly on bone cement. Treatment with the supernatants of NAC-containing bone cement did not lead to release of proinflammatory IL-6, but reduced cell viability and proliferation at higher concentrations. NAC treatment also resulted in slight changes in p38 activation compared to medium. Therefore, effectiveness of NAC against PJI-related pathogens could be demonstrated, but used concentrations need to be chosen with care to avoid cytotoxic effects.

*S*. *pseudintermedius* was chosen to focus on in this study because together with *S. aureus*, this pathogen is responsible for more than 50% of PJIs in small animals (Hayes et al., 2013). It is also becoming more relevant as a zoonotic pathogen especially in humans in close contact with small animals (Van Hoovels et al., 2006; Moses et al., 2023). With broth microdilutions, a MIC of 2.5 mg/mL NAC was determined for the three clinical isolates used in this study. These results go in line with MICs of NAC determined with other isolates of *S. pseudintermedius* in our lab (Walter et al., 2023). For *Staphylococcus epidermidis*, *Klebsiella pneumoniae*, and *P. aeruginosa*, which are further pathogens that can cause PJIs, MICs of 4–5 mg/mL have been determined (Gomes et al., 2012; Young et al., 2019; De Angelis et al., 2022).To incorporate NAC into PMMA bone cement, higher amounts of NAC with 50 mg and 100 mg were chosen to ensure even distribution of NAC in the cement mass and release of substance from the cement surface. We hypothesized that these concentrations of NAC would prevent *S. pseudintermedius* from attaching to the cement or kill bacteria growing on the surface. Live/Dead staining revealed that NAC prevented attachment of bacteria to the cement but could not completely inhibit colonization (Figure 1). For a complete inhibition, even higher amounts of NAC could be necessary, or NAC could be incorporated together with an antibiotic. Valzano et al. (2022) demonstrated a synergistic effect of NAC together with the antibiotic colistin against *Pseudomonas aeruginosa* biofilms, while Manoharan et al. (2020) found that a combined treatment with NAC, amoxicillin/clavulanate and amylase significantly decreased viability of *S. aureus* biofilms. Furthermore, novel formulation techniques, e.g., incorporating NAC into microspheres before mixing it into the cement could change the substance release profile to a more efficient and controlled release (Spicer et al., 2013).

To ensure cell compatibility of bone cement with NAC, several experiments concerning the viability, proliferation, cytokine release and stress-related kinase activation were performed. After treatment of HOS cells with NAC diluted in cell culture medium, the IC_50_ for viability was 3.6 mg/mL and proliferation was reduced to less than 70% of control at 4 mg/mL (Figure 2). These concentrations are above the MIC of 2.5 mg/mL determined with the *S. pseudintermedius* isolates, so that bacterial growth can be inhibited while avoiding cytotoxicity. In literature, different tolerable concentrations of NAC are found in *in vitro* settings depending on the cell type. NAC did not have a cytotoxic effect on bladder epithelial cells at concentrations up to 8.16 mg/mL in the first 24 h (Manoharan et al., 2021), while human osteoblasts could tolerate NAC at up to 2.3 mg/mL (Tsukimura et al., 2009). The results presented here seem to be comparable to those described for human osteoblasts (Tsukimura et al., 2009). Growth of bone cells on and around the bone cement is essential for integration of the implant. Seeded directly onto bone cement containing NAC, attachment of HOS cells to the cement and viability of cells growing on the cement were analyzed after 24 h (Figure 3). Interestingly, total cell number per mm^2^ was reduced on bone cement with 50 mg NAC compared to control, while on bone cement with 100 mg NAC, total cell number was comparable to control, but all attached cells were dead. A possible scenario is that 100 mg NAC released into the medium immediately kills HOS cells before attachment to the cement so that dead cells sink down onto the cement. 50 mg NAC does not seem to be immediately cytotoxic but inhibits attachment to the cement or leads to detachment of cells. However, because several washing steps are performed to remove detached cells before staining, further research is necessary to explain the difference in cell numbers. At lower concentrations, NAC can enhance cytocompatibility of PMMA bone cement due to its antioxidative properties (Tsukimura et al., 2009). The concentrations chosen here seem to be too high to exhibit this effect. To simulate flow of tissue fluid around the implant and the release profile of substances from bone cement, bone cement platelets with NAC and without additive were incubated in four different volumes of medium and HOS cells were treated with the supernatants (Figure 4). The most substance is released from bone cement in the hours after implantation, so that this early phase is a critical point for viability and proliferation of surrounding cells. The supernatants with higher concentrations of NAC, particularly of bone cement with NAC incubated in 1 mL and 3 mL medium, reduced the viability and proliferation of HOS cells. Proliferation was more strongly affected by the treatment, with significant reduction also by 10 mL and 30 mL supernatants. Because proliferation of cells surrounding the implant is critical for regeneration, it is important to keep in mind that proliferation seems to be more sensitive towards treatment. In viability testing of bone cement with up to 50% NAC, Sukhonthamarn et al. (2021) found that bone cement without additive had higher cytotoxicity than cement with NAC. Here, we could observe reduction of proliferation by bone cement without additive, but not reduction of viability, and cytotoxic effects were caused by bone cement with NAC.

Furthermore, the aim was to test if bone cement together with NAC would cause sterile inflammation and release of cytokines in HOS cells. IL-6 is a multifunctional cytokine regulating inflammatory responses and also osteoclastogenesis and bone resorption (Guo et al., 2018). It has also been shown that IL-6 release can be caused by a reaction to particles of PMMA bone cement (Gonzalez et al., 1996). In this study, IL-6 was only released from HOS treated with LPS or PGN, not from the cells treated with bone cement supernatants (Figure 4C). Interestingly, IL-6 is also considered as a marker of PJIs and can be used as a diagnostic tool (Randau et al., 2014). NAC has also been shown to inhibit IL-6 release caused by LPS stimulation at 0.16 mg/mL (Guo et al., 2018) but since NAC concentrations were much higher in this study, this does not explain why no IL-6 was released. From these results, we conclude that treatment with NAC-containing bone cement supernatants does not lead to signs of sterile inflammation. The pathway of p38 mitogen-activated protein kinase (MAPK) is involved in downstream regulation of cell cycle, apoptosis and inflammation following diverse stress stimuli, e.g., UV radiation or heat (Zarubin and Han, 2005). Activation of the p38 pathway is also involved in osteoclastogenesis provoked by PMMA particles (Abbas et al., 2003). For this reason, the influence of NAC and NAC-containing bone cement supernatants on the levels of p38 and activated p38 (p-p38) were analyzed by Western blotting (Figure 5). On average, treatment with NAC or NAC-containing bone cement supernatants led to lower p38 and higher p-p38 levels, indicating an activation of p38 following the treatment. Significant differences in p38 and p-p38 levels were only found between medium and 5 mg/mL NAC treatment, but it must be kept in mind that 5 mg/mL NAC is cytotoxic for HOS cells, which influences general protein expression. Treatment with the supernatant of bone cement without additive led to similar levels of p38 and p-p38 as in treatment with bone cement and NAC. In cells of the airways, NAC treatment has been found to reduce p38 activation and cytokine release by providing intracellular glutathione (Hashimoto et al., 2001; Wuyts et al., 2003). In HOS cells, involvement of the p38 pathway has been examined mostly in the context of anti-tumor treatments. For example, p38 is activated in HOS cells after treatment with the anti-tumor and anti-inflammatory substance Licochalcone A resulting in apoptosis (Lin et al., 2019), while capsaicin inactivates the p38 pathway (Zhang et al., 2017). To conclude, it could be shown that NAC and bone cement together with NAC could change p38 activation, although this overall effect was not statistically significant.

In infection experiments, the effect of NAC at a concentration below the MIC on *S. pseudintermedius* internalization into and adhesion to HOS cells was investigated (Figure 6). A concentration below the MIC was chosen for this purpose due to the release profile of substances from PMMA bone cement with low and stable release after a first burst (Sun et al., 2021). It has been shown before that manifestation of *S. pseudintermedius* joint and bone infection is likely from its ability to invade non-professional phagocytes like osteoblasts (Maali et al., 2016). Inhibition of bacterial adhesion and invasion of bladder epithelial cells by NAC was proven with *E*. *coli* and *Enterococcus faecalis* (Manoharan et al., 2021). Here, a similar effect could not be shown with HOS cells and *S. pseudintermedius*. NAC did not reduce internalization or adhesion compared to medium.

One limitation of this study was that we did not measure the exact amount of NAC released from each platelet, so that the exact NAC concentrations in supernatants are not known and are not completely comparable to concentrations of pure NAC. In addition, further experiments including different types of bone cells and additional testing of antibacterial activity including a possible development of resistances against NAC could have provided wider knowledge about the suitability as an additive to bone cement. Since this study concentrated on the suitability of NAC as an active substance, stability of bone cement with added NAC was not considered here.

## 5 Conclusion

It could be shown that NAC is effective against PJI-relevant *S. pseudintermedius* at non-cytotoxic concentrations for human derived bone cells. At a concentration below the MIC, infection of cells could not be prevented. Together with bone cement, reduced viability and proliferation, cell death and impaired attachment could be observed after some of the NAC treatments. There was no release of proinflammatory IL-6, but the results indicate possible involvement of the p38 MAPK pathway in stress reactions. The present data suggest that at the tested concentrations, NAC is not yet suitable for the use with bone cement due to limited cytocompatibility. Thus, further research is required to enhance biocompatibility, e.g., due to novel release methodologies. Measurement of exact NAC release from bone cement to balance cytocompatibility and antibacterial effect as well as combination of NAC with established antibiotics to determine a possible synergistic effect are options for follow-up research.

## 6 Impact statement

Periprosthetic joint infections after endoprosthetic surgery are a serious threat with devastating impacts on patients. The rising occurrence of multidrug resistant pathogens complicates the prevention and treatment of such infections; therefore, alternative treatment strategies are needed. This study provides an evaluation of NAC as a prevention and treatment option for the use together with bone cement. An emphasis is laid on biocompatibility to assess the influence on post-surgical regeneration. The results indicate that the chosen concentration of NAC is critical to balance biocompatibility and antibacterial effectiveness. With the presented results, a foundation for further research concerning clinical use of NAC as a strategy against periprosthetic joint infections is provided.

## Data Availability

The raw data supporting the conclusions of this article will be made available by the authors, without undue reservation.
