# Supplementary material for: N-acetyl cysteine as an additive to bone cement against pathogens involved in periprosthetic joint infections
Source: Front Bioeng Biotechnol. 2025 Aug 28;13:1595821. doi: 10.3389/fbioe.2025.1595821 (PMC12423035; doi:10.3389/fbioe.2025.1595821)
Supplement: Supplementary file 1 [file DataSheet1.pdf]

Supplemental Figure 1: Uncropped Western Blots

Figure 5A: p38; NAC in mg/mL

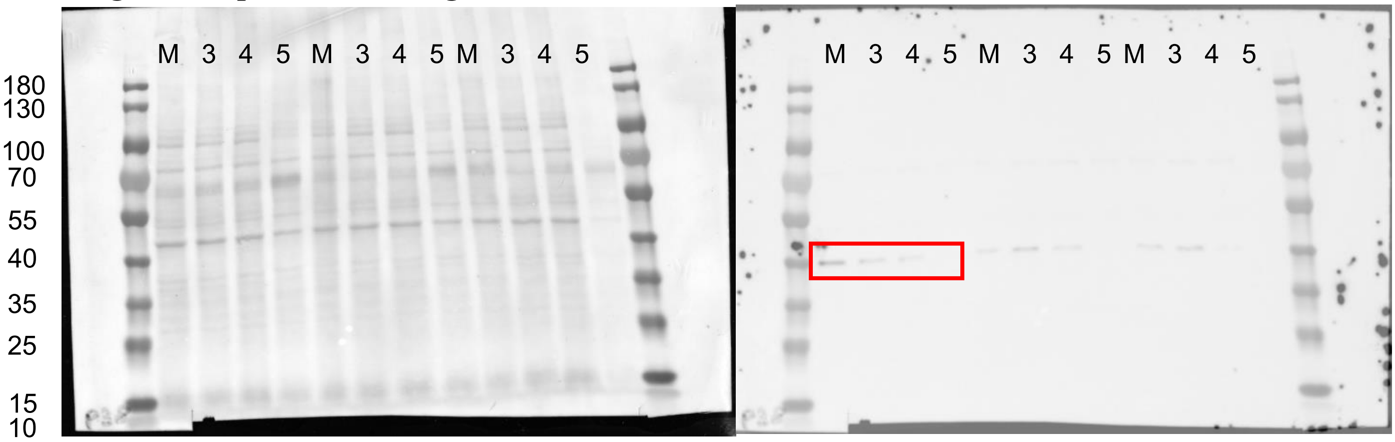

Figure 5B: p-p38; NAC in mg/mL

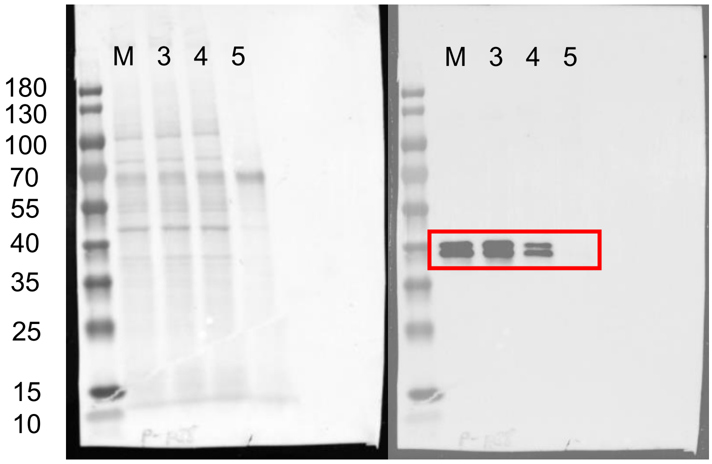

Figure 5C: p38; NAC in bone cement in mg

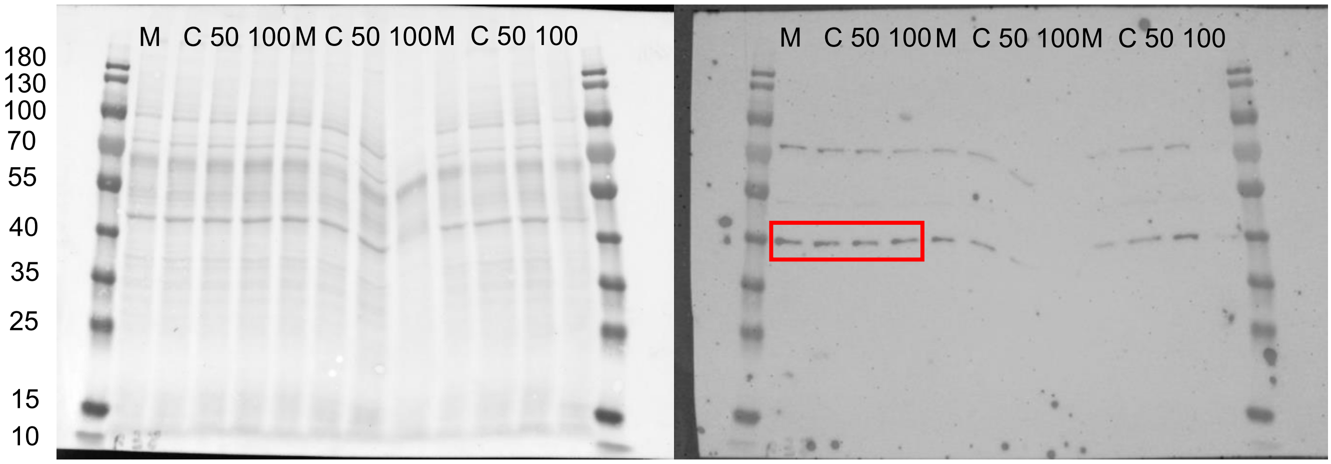

Figure 5D: p-p38; NAC in bone cement in mg

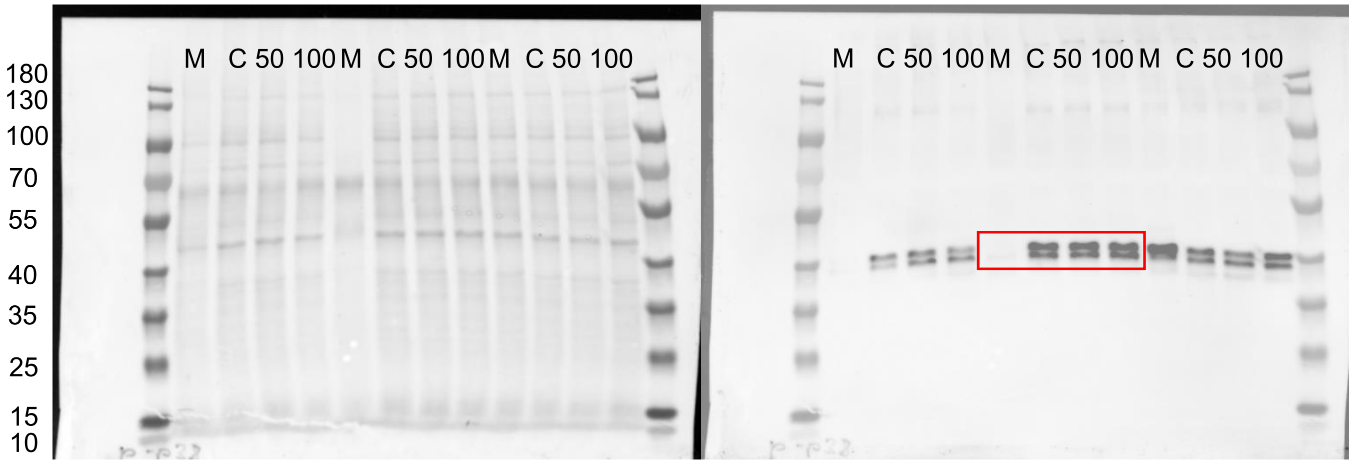

Figure S1: Full blots of cropped images shown in Figure 5. Left: visualized with Ponceau S red staining and used for normalization. Right: specific protein as indicated visualized with chemiluminescence. Protein size is expressed in kDa. Red boxes highlight the bands depicted in the manuscript. Concentrations of pure NAC are shown in mg/mL (3, 4, and 5 mg/mL), amount of NAC per bone cement platelet is shown in mg (50 and 100 mg). M = medium; C = control, bone cement without additive.

Supplemental Figure 2: Preparation of bone cement platelets

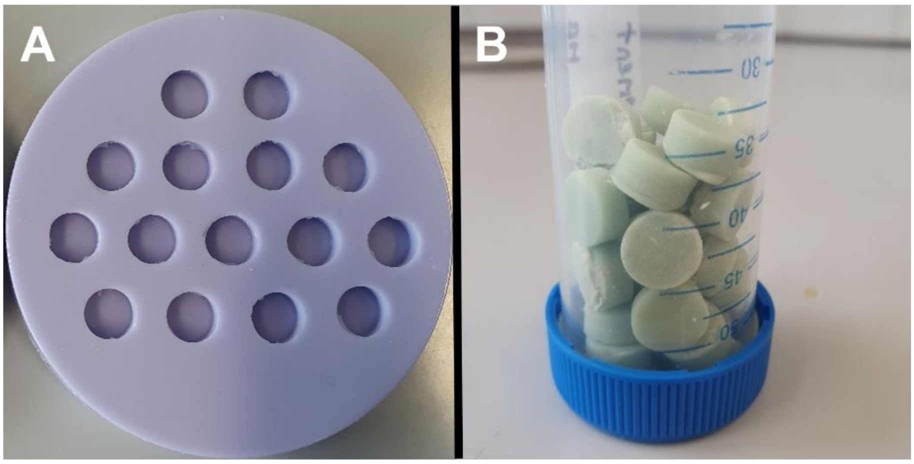

Figure S2: Preparation of bone cement platelets as described in Section 2.3. **(A)**: Silicone mold used to form platelets with a diameter of 1 cm. **(B)**: Hardened platelets stored in a sterile falcon tube.
